# Multi-component school intervention reduces obesity and improves health behaviors in children: a cluster- randomized controlled trial

**DOI:** 10.1038/s41598-025-24295-y

**Published:** 2025-11-18

**Authors:** Peifen Duan, Chen Li, Zhenxing Yuan, Jianhui Yuan, Xiangxian Feng

**Affiliations:** 1 School of Public Health, Changzhi Medical College, Changzhi, 046000 China; 2 Laboratory of Environmental Factors and Population Health, Shanxi Higher Education Institutions of Science and Technology Innovation Plan Platform, Changzhi, 046000 China; 3 Key Laboratory of Environmental Pathogenic Mechanisms and Prevention of Chronic Diseases, Changzhi Medical College, Changzhi, 046000 China; 4 School of Nursing, Changzhi Medical College, Changzhi, 046000 China

**Keywords:** Childhood obesity, Comprehensive intervention, Effectiveness evaluation, Public health, Weight management, Medical research

## Abstract

Childhood obesity has become a global health priority due to its escalating prevalence and associated health risks. We conducted a cluster-randomized controlled trial to assess the impact of a comprehensive school-based intervention on obesity-related outcomes among primary school children in China. Eight schools (*n* = 400 students, 396 completed) were randomly allocated (1:1) to intervention or control groups during the 2018–2019 academic year. The 10-month intervention incorporated a structured health education curriculum, enhanced physical activity sessions, periodic body mass index (BMI) monitoring with personalized feedback, and family engagement activities as its core components.  The primary outcome was change in BMI. While secondary outcomes encompassed obesity prevalence, body composition, dietary behaviors, physical activity, and health knowledge. The analysis revealed significantly greater reduction in BMI in the intervention group compared to controls (mean difference: -0.36 kg/m²; 95% CI: -0.58 to -0.13; *P* = 0.002). Subgroup analyses revealed differential effects by sex, father’s educational level, overweight or obesity at baseline, and main caregivers (parents) (all *P* < 0.05), whereas, no evidence of difference across the main caregivers (non-parents) and the mother’s education level (above high school). Obesity prevalence decreased by 3.54% in the intervention versus 0.51% in control schools (OR = 0.34, 95% CI: 0.25 to 0.45; *P* = 0.020). Significant improvements were noted in obesity-related knowledge, dietary habits, moderate-to-vigorous physical activity (MVPA), and reduced sedentary time (all *P* < 0.05), though fitness measures showed no significant between-group differences. Overall, these findings demonstrated that a well-designed school-based program can effectively mitigate obesity risk and promote healthier behaviors among children. Further research should examine long-term sustainability and scalability across diverse settings.

**Supplementary Information:**

The online version contains supplementary material available at 10.1038/s41598-025-24295-y.

## Introduction

The global epidemic of childhood obesity continues to escalate, posing unprecedented challenges to public health systems worldwide^1^. Current estimates indicate that nearly one-quarter (22%) of children and adolescents aged 5–19 years exceed healthy weight standards, representing approximately 435 million individuals globally^2,3^. Alarmingly, epidemiological projections forecast this prevalence could approach 40% by 2035, potentially affecting 770 million young people if current trends remain unaddressed^2,4^. Although it is a fact that the rise in overweight and obesity has generally leveled off in many developed countries^1,5^, recent data indicate that obesity among school-age children in developing countries is still increasing^6–8^. Especially in China, where rapid socioeconomic development and nutritional transitions have contributed to increasing pediatric adiposity^9,10^. National surveillance data indicate that 11.1% and 7.9% of Chinese children and adolescents meet the criteria for overweight and obesity, respectively^11^, with models predicting these combined rates may reach 28% by 2030 without effective countermeasures.

The consequences of pediatric obesity extend beyond childhood, elevating risks for subsequent cognitive impairment, mental health disorders, and cardiovascular disease^12–14^. This complex condition arises from multi-factorial determinants including genetic predisposition, dietary patterns, and physical inactivity, necessitating comprehensive prevention strategies targeting schools, families, and communities.

Existing evidence for school-based interventions remains inconclusive. While some systematic reviews demonstrated modest improvements in body mass index (BMI) and health behaviors through comprehensive school programs^15–18^, other studies reported limited effects on adiposity outcomes^19–21^. These discrepancies may stem from methodological limitations, including heterogeneous intervention designs, brief follow-up durations, and inconsistent implementation fidelity. Notably, most evidence has been derived from Western populations, with scarce data from Chinese settings. Family involvement has shown promise in augmenting intervention efficacy^22^, though combined lifestyle programs incorporating both dietary and exercise components have yielded inconsistent results regardless of parental participation^23^.

Based on the above issues, we implemented a cluster-randomized controlled trial in Northern China’s primary school system. The study evaluated the efficacy of a novel, multi-component intervention strategy targeting childhood obesity prevention through synergistic school-family partnerships.

## Materials and methods

### Study design and participants

This cluster-randomized controlled trial was conducted in 8 primary schools in Changzhi, Shanxi Province, China, between September 2018 and June 2019. Using the random cluster sampling method, we selected one grade 4 class from each participating school. All participants and their legal guardians provided written informed consent before enrollment. The study protocol received ethical approval from the Institutional Review Board of Peking University (IRB00001052-18021). This trial was registered at the Chinese Clinical Trial Registry (registration number: NCT03665857) on 11 September 2018. This research complied with the Declaration of Helsinki. This trial was documented in accordance with the CONSORT 2010 guidelines.

### Randomization

Following baseline assessments, 8 participating schools were randomly allocated (1:1) to either intervention or control arms through a centralized computer-generated randomization process. An independent statistician maintained concealed allocation sequences to ensure blinding integrity.

### Intervention

This school-based intervention, grounded in the social-ecological model, was implemented over one academic year (September 2018 to June 2019) and comprised five key components. Three components of the intervention targeted the children (1) health education: Primary school teachers were trained by the project staff and delivered the health education every 2–3 weeks for 10 sessions. The core messages were “do not overeat; avoiding sugary drinks; reducing intake of high energy foods; minimizing sedentary time and increasing exercise.” (2) physical exercise reinforcement: Certified physical education teachers supervised daily 60-minute moderate-to-vigorous physical activity (MVPA) sessions, and (3) BMI monitoring and feedback: Trained teachers measured the children’s height and weight monthly, with results and trends communicated to students and parents via a mobile application. The teacher would also measure the child’s weight and give feedback to every child weekly. Two components targeted the children’s environment (1) schools: The intervention schools implemented obesity-related policies such as no selling, consuming, or purchasing of unhealthy snacks or sugar-sweetened beverages on campus. Curriculum time is available for health and exercise in schools. (2) families: Parents received three in-person health education courses including 5 core messages (2 no, 2 less, 1 more). They were encouraged to reinforce healthy dietary habits and physical exercise in home settings, with instruction to utilize the mobile application for behavioral tracking and progress monitoring. Detailed information were provided in Table S1 (Supplementary Materials).

Control schools conducted their usual health education curriculum and physical activity classes.

### Follow-up and outcomes

Follow-up was conducted at the end of the first and second semesters after randomization. The staff who implemented the interventions did not participate in the follow-up assessments. The principal outcome was the change in BMI between baseline and trial completion. Secondary outcomes included: body composition (percentage body fat, waist circumference, and waist-to-hip ratio), weight status, dietary behaviors and physical exercise and obesity-related knowledge.

Weight status was primarily evaluated based on BMI, calculated as weight in kilograms divided by the square of height in meters (kg/m²). Participants were categorized into the following groups: underweight, normal weight, overweight, and obesity. Overweight and obesity were classified according to the Chinese Screening Criteria for Overweight and Obesity in School-Aged Children and Adolescents^24^, using age- and sex-specific BMI cut-off values.

### Data collection

Before and after the intervention, physical examinations and questionnaire surveys were conducted by uniformly trained investigators. Key measurements included anthropometric measurements (height, weight, waist and hip circumferences, and body fat percentage) and physical fitness evaluation (standing long jump, rope jumping, shuttle run, and sit-up). The questionnaire covered: demographic characteristics (gender, age); knowledge related to diet and physical activity; dietary and exercise behaviors and attitudes. Assessment tools, measurement procedures, and corresponding results were provided in Table S2 in Supplementary Material.

### Sample size calculation

Power analysis was conducted assuming a between-group difference of 0.50 BMI units (SD = 1.40) with 10% expected dropout. The calculated sample size of 400 participants (α = 0.05, 1-β = 0.88, two-sided) was obtained through cluster sampling across 8 schools, yielding an average cluster size of 50 children.

### Statistical analysis

Data were analyzed using SPSS version 26.0 (IBM Corp). The primary outcome analysis included children with complete BMI data collected at both baseline and the end of the trial. Participants with missing end-of-trial BMI data (*n* = 4, 1.0%) were excluded based on a predefined criterion of a missing rate < 5%. Continuous variables following a normal distribution are expressed as mean ± standard deviation (SD), whereas those deviating from normality are summarized using median and interquartile range (IQR). Categorical variables are expressed as frequencies and percentages. To evaluate intervention effects on primary and secondary outcomes, generalized estimating equation (GEE) models were employed, accounting for potential confounding variables including sex, baseline age, and weight status. Subgroup analyses were conducted to examine potential effect modification by sex, baseline weight status, maternal education level, paternal education level, and single-child status. All statistical tests were two-tailed, with significance set at *P* < 0.05.

## Results

### Study implementation and baseline characteristics

**Fig. 1:**
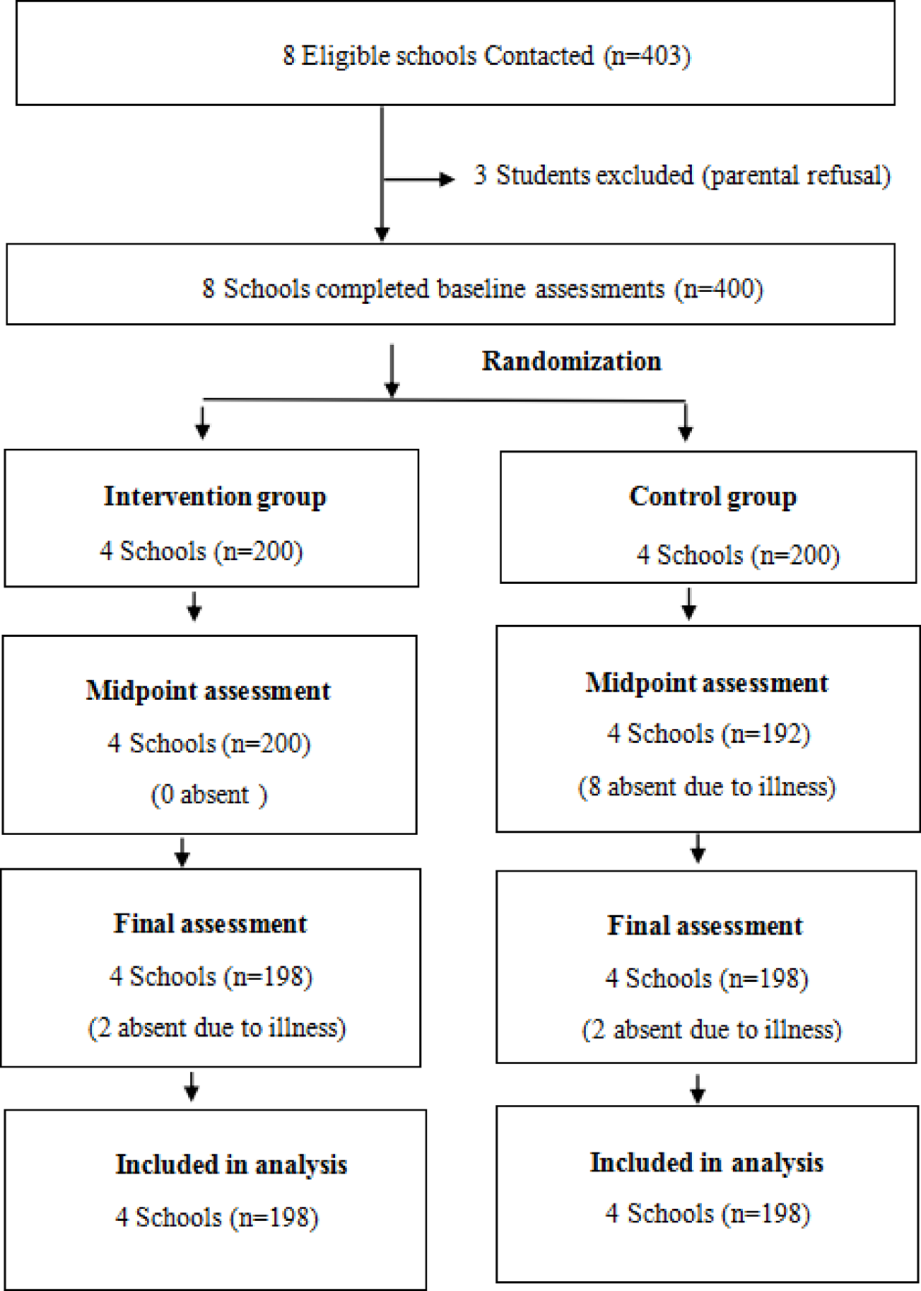
Participant enrollment and allocation flowchart.

The participant enrollment process is summarized in Fig. 1. Initially, 400 fourth-grade students from eight recruited schools were enrolled in the baseline assessment. During the study period, four students (two from each group) were lost to follow-up due to illness, resulting in a final sample of 396 subjects (198 per group), with equal gender distribution (198 boys and 198 girls). The study population had a mean age of 9.14 ± 0.38 years and a mean BMI of 18.3 ± 3.59 kg/m² at baseline. Demographic and anthropometric characteristics were well-balanced between study arms, except for age (*P* = 0.025) and sex distribution (*P* = 0.044), which showed statistically significant (Table 1). A comparison of baseline characteristics between children included in the primary outcome analysis (*n* = 396) and those lost to follow-up (*n* = 4) demonstrated no significant differences (Table S3, Supplement Material).

**Table 1:** Baseline demographic and anthropometric characteristics of study participants.

Characteristic	Intervention(*n* = 198)	Control (*n* = 198)	Statistical value	*p*
Sex (boys), No (%)	89(44.95)	109(55.05)	4.040	**0.044**
Primary caregiver (parents), No (%)	190(95.96)	185(93.43)	1.257	0.262
Maternal educational level (above high school), No (%)	107(54.04)	98(49.49)	0.644	0.422
Weight status (overweight/obesity), No (%)	76(38.38)	87(43.94)	1.841	0.606
Age, mean (SD), y	9.18(0.42)	9.10(0.33)	2.253	**0.025**
Height, mean (SD), cm	138.25(6.47)	138.27(6.24)	−0.032	0.975
Weight, mean (SD), kg	35.06(8.83)	35.74(8.69)	−0.766	0.444
BMI, mean (SD), kg/m^2^	18.20(3.61)	18.54(3.56)	−0.948	0.344
Waist circumference, mean (SD), cm	64.12(9.51)	63.79(12.02)	0.309	0.757
Hip circumference, mean (SD), cm	75.57(7.70)	74.14(11.15)	1.478	0.140
Body fat percentage, mean (SD)	19.33(10.72)	19.94(10.71)	−0.571	0.569

Categorical variables were presented as No (%), and between-group comparisons were performed using the ***χ²*** test. Continuous variables conforming to a normal distribution were expressed as mean (SD), and between-group comparisons were analyzed using the independent samples ***t*** test.

### Effect on the primary outcome

**Fig. 2:**
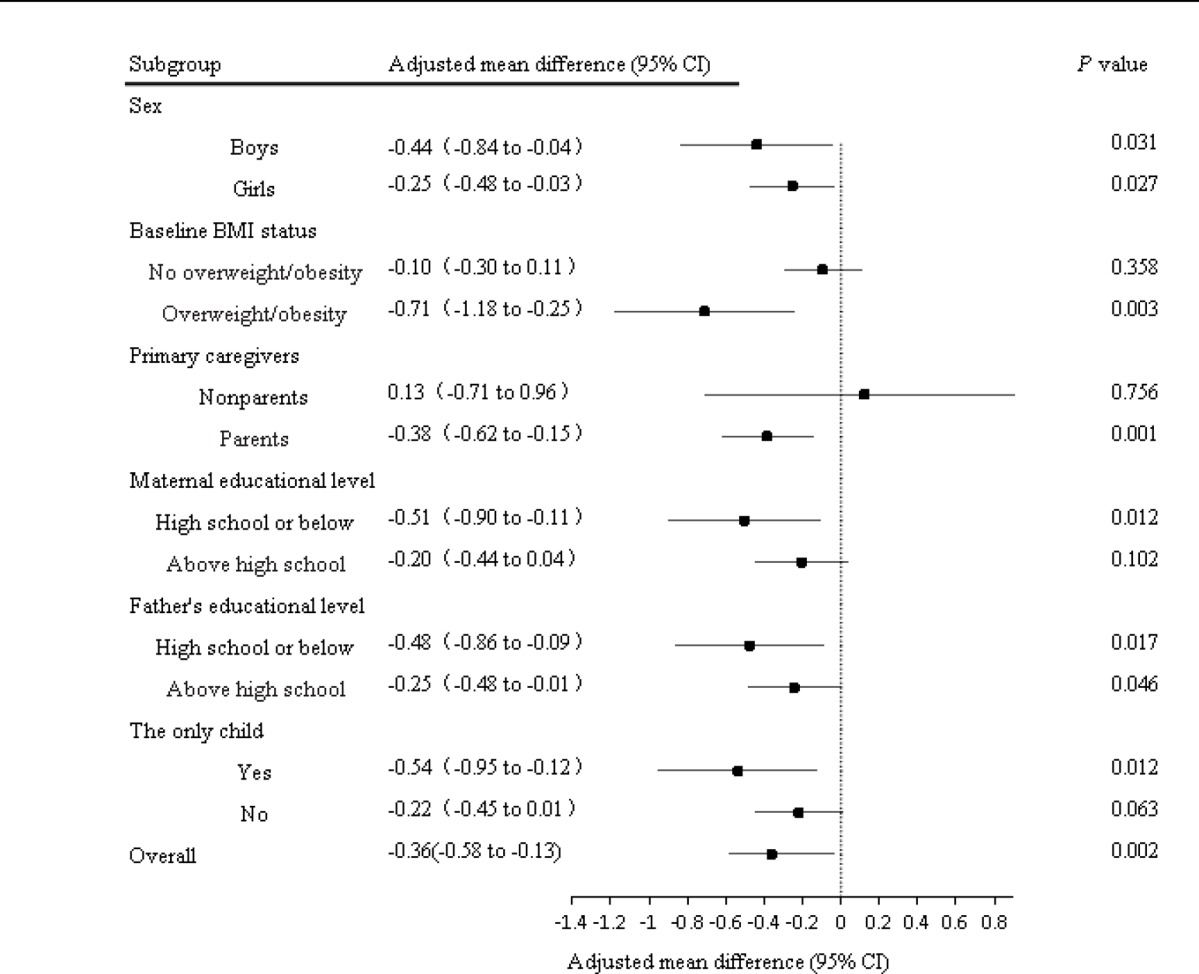
Differential intervention effects on BMI across prespecified subgroups.

Following multivariable adjustment for sex, baseline age, and weight status, participants in the intervention arm exhibited significantly greater BMI reduction relative to controls (adjusted mean difference: −0.36 kg/m²; 95% CI: −0.58 to −0.13; *P* = 0.002). Subgroup analyses revealed differential effects across population strata. The intervention demonstrated greater BMI reduction in boys versus girls, participants with paternal education ≤ high school versus > high school, and baseline overweight/obese children (all *P* < 0.05). Similarly enhanced effects were observed for children with parental caregivers, maternal education ≤ high school, and single-child families (all *P* < 0.05). No significant effects were detected in subgroups with non-parental caregivers (*P* = 0.756), highly educated mothers (*P* = 0.102), or multiple children (*P* = 0.063). (Table 2; Fig. 2).

**Table 2:** Effects of multilevel interventions on BMI [mean (SD), kg/m^2^].

No. intervention/control	Intervention group		Control group		Mean difference 95%CI	*P*
	Baseline	Endpoint	Baseline	Endpoint		
198/198	18.20(3.61)	18.14(3.62)	18.54(3.56)	18.83(4.11)	−0.36(−0.58, −0.13)	**0.002**

### Effects on the secondary outcomes

The intervention demonstrated significant improvements across multiple secondary endpoints (Table 3). Anthropometric measures showed greater reductions in the intervention group for waist circumference (−3.48 cm; 95% CI: −4.70 to −2.25; *P* = 0.001) and waist-to-hip ratio (−0.02; 95% CI: −0.03 to −0.01; *P* = 0.001), though body fat percentage changes were non-significant (*P* = 0.059). Obesity prevalence decreased more substantially in the intervention versus the control group (OR = 0.34, 95% CI: 0.25–0.45; *P* = 0.020). Similarly, the prevalence of overweight/obesity in the intervention group also decreased compared to the control group (OR = 0.82, 95% CI: 0.70–0.96; *P* = 0.015).

**Table 3:** Effects of multilevel interventions on study outcomes.

Outcomes	No. intervention/ control	Intervention group		Control group		Mean difference/ OR 95%CI	*p*
		Baseline	Endpoint	Baseline	Endpoint		
Obesity-related index, mean (SD)							
Body fat percentage	198/198	19.33(10.72)	16.73(9.40)	19.94(10.71)	18.22(10.03)	−0.88(−1.79,0.03)	0.059
Waist circumference, cm	198/198	64.12(9.51)	61.75(9.85)	63.79(12.02)	64.89(10.71)	−3.48(−4.70,−2.25)	0.001
Waist-to-hip ratio	198/198	0.85(0.05)	0.81(0.06)	0.86(0.05)	0.85(0.07)	−0.02(−0.03,−0.01)	0.001
Weight status, No (%)							
Obesity*	198/198	43(21.72)	36(18.18)	51(25.76)	50(25.25)	0.34(0.25,0.45)	0.020
Overweight/obesity*	198/198	76(38.38)	67(33.84)	87(43.94)	78(39.39)	0.82(0.70,0.96)	0.015
Dietary behaviors and physical activity							
Not eating fried food, No (%)	198/198	95(47.98)	128(64.65)	92(46.46)	72(36.36)	1.83(1.39,2.42)	< 0.001
Not eating western fast food, No (%)	198/198	159(80.30)	181(91.41)	157(79.30)	154(77.78)	1.64(1.15,2.34)	0.006
Taking weight loss action, No (%)	198/198	112(56.57)	191(96.46)	97(48.99)	148(74.75)	9.03(3.98,20.52)	< 0.001
Screen time < 2 h/d, No (%)	198/196	119(60.10)	146(73.74)	126(64.29)	116(59.19)	1.94(1.27,2.96)	0.002
Daily MVPA ≥ 1 h, mean (SD), d/w	198/198	2.61(1.99)	4.57(2.44)	2.37(1.87)	4.08(2.60)	0.38(0.07,0.70)	0.017
Obesity-related knowledge, mean (SD), core point							
Obesity-related knowledge	198/198	6.64(1.48)	7.32(1.10)	6.76(1.28)	7.08(1.09)	0.37(0.08,0.65)	0.012
Physical fitness, mean (SD)							
Distance of long-standing jump, cm	184/197	136.26(19.00)	144.31(18.64)	135.80(18.16)	141.64(20.41)	1.57(−1.56,4.69)	0.326
No. of rope jumps within 1 min	188/197	79.75(32.41)	108.79(30.70)	83.10(33.44)	90.56(30.47)	6.95(2.13,11.78)	**0.005**
Duration of shuttle run (50 m×8), s	188/196	128.92(21.72)	120.16(18.04)	130.46(23.54)	120.47(32.77)	−1.10(−4.56,2.36)	0.533
No.of sit-ups within 1 min	188/197	23.25(11.64)	31.18(8.35)	24.49(11.42)	30.62(11.72)	−0.24(−1.98,1.50)	0.784

*Obesity was defined as the number of children meeting the obesity criteria specified by Chinese national screening standards. Overweight/obesity was defined as the total number of children meeting the criteria for either overweight or obesity according to the same guidelines.

Significant between-group differences were observed in dietary behavior [not eating fried food (OR = 1.83, 95% CI: 1.39–2.42; *P* < 0.001), not eating western fast food (OR = 1.64, 95% CI: 1.15–2.34; *P* = 0.006), taking weight loss action (OR = 9.03, 95% CI: 3.98–20.52; *P* < 0.001)] and physical activity [screen time < 2 h/d (OR = 1.94, 95% CI: 1.27–2.96; *P* = 0.002), daily MVPA ≧ 1 h (MD: 0.38 days, 95% CI: 0.07–0.70; *P* = 0.017)]. The intervention group showed greater improvement in obesity-related knowledge (MD: 0.37 points; 95% CI: 0.08–0.65; *P* = 0.012). Physical fitness measures remained comparable between groups except for rope jumping performance (MD: 6.95; 95% CI: 2.13–11.78; *P* = 0.005).

## Discussion

This school-clustered RCT evaluated the effectiveness of a multi-component intervention for preventing obesity among children aged 8–10 years. Following one academic year of intervention, participants demonstrated significant reductions in both mean BMI and obesity prevalence. The intervention additionally improved secondary adiposity markers (e.g.: waist circumference, waist-to-hip ratio), sedentary behaviors, and obesity-related knowledge. However, no significant effects were observed for body fat percentage or physical fitness.

The global prevalence of childhood obesity continues to rise, yet progress in corresponding preventive interventions remains limited. School-based interventions are important to prevent childhood obesity. Recent meta-analysis confirmed the efficacy of school-delivered obesity prevention programs in reducing BMI among children^24,25^. Our findings demonstrated that the BMI of the intervention group decreased by −0.36 kg/m^2^ compared to controls (95%CI: −0.58 to −0.13; *P* = 0.002) after one academic year of school-based obesity intervention from a family and individual perspective. This contrasts with two UK cluster RCTs that reported null effects on BMI^26,27^, possibly due to differences in intervention design. Evidence from multiple clinical studies demonstrated that multimodal lifestyle interventions (such as physical activity, diet, and behavior changes) can effectively mitigate childhood obesity^28–30^. A systematic review of 26 Chinese interventions found comprehensive programs incorporating health education were more effective than physical activity alone^31^. The positive outcomes observed in our study may be attributed to its comprehensive, multi-component design, which integrated health education, regular BMI monitoring, and the use of mobile health technology. This integrated approach likely fostered a conducive environment for behavioral change. Furthermore, the design required and may have facilitated a degree of parental involvement, such as participation in educational sessions and use with the app, which may also have contributed to its effectiveness.

Subgroup analyses revealed significant gender differences in intervention efficacy, with boys demonstrating greater BMI reduction than girls (−0.44 vs −0.25 kg/m²), which aligns with the results of a systematic review^32^, but contrasts with a Slovenian school-based intervention showing stronger effects in girls^33^. The systematic review showed that higher parental education was associated with lower obesity rates^34^, while our study showed that children with lower parental education had a greater effect on BMI. The possible reason is that parents with less than high school may not have regular jobs and may have more time to accompany and supervise their children. Our intervention also resulted in significant reductions in waist circumference and waist-to-hip ratio (both *P* = 0.001), which was partially consistent with a Victorian secondary school study that reported waist circumference reductions in boys alongside decreased sugar-sweetened beverage consumption, though no significant effects were observed in girls^35^.

Nutrition literacy has been identified as a modifiable determinant of obesity-related behaviors in youth^36,37^, with China Health and Nutrition Survey (CHNS) data confirming its protective association against excess weight^38^. Our intervention achieved 0.37 scores improvement (95% CI: 0.08–0.65; *P* = 0.012) in obesity-related knowledge compared to controls, consistent with findings from a nationwide Chinese school-based program that documented enhanced health literacy among children aged 6–18 years^39^. However, unlike our study, that intervention did not observe significant changes in dietary or physical activity behaviors, possibly due to the short duration of the intervention. It was reasonable that behavioral modifications often require longer observation periods and more intensive interventions to yield measurable effects. Physical activity promotion is widely recognized as an effective strategy in pediatric weight management^40^. Multiple studies have reported an inverse association between MVPA and adiposity^41–44^. Our study revealed a statistically significant between-group difference in MVPA engagement, with the intervention group demonstrating superior improvement (mean difference = 0.38 days, 95% CI: 0.07–0.70; *P* = 0.017). This observation suggests the implemented intervention successfully promoted MVPA participation among study participants. The observed enhancement in MVPA may exert beneficial effects on obesity-related parameters through various physiological pathways, including enhanced energy expenditure and improved metabolic regulation^45,46^. Further investigations are warranted to elucidate potential synergistic effects between MVPA and other modifiable lifestyle factors, which could inform the development of more effective intervention approaches.

### Strengths and limitations

#### Strengths

This study has several notable strengths. First, we implemented a comprehensive, school- and child-centered intervention combining health education, structured physical activity, BMI monitoring with feedback, and family engagement strategies. Additionally, supportive school policies were introduced to reinforce behavioral changes in intervention schools. Second, randomization was conducted after baseline assessments, and outcome assessors were blinded to group allocation, minimizing measurement bias. Third, the study maintained a high retention rate, with minimal loss to follow-up. Fourth, the intervention was grounded in a well-established theoretical framework^47^, enhancing its potential effectiveness compared to non-theory-based approaches.

### Limitations

Several limitations should be considered when interpreting our findings. First, although a standardized intervention protocol was implemented, the lack of systematic assessment of implementation fidelity—including teachers’ adherence to curriculum delivery and parent attendance rates—may have introduced variability in intervention delivery across schools. This could potentially attenuate the observed intervention effects. Second, although educational materials and guidance were provided to families, the absence of quantitative evaluation of parental reinforcement of healthy behaviors at home limits our understanding of the intervention’s mechanism of action. Third, the use of self-reported obesity-related behavioral data from children or guardians may be subject to recall and social desirability biases, likely resulting in non-differential misclassification that would bias effect estimates. Fourth, physical activity metrics were based solely on subjective reports without objective validation, potentially reducing measurement accuracy. Future studies should prioritize the refinement of implementation fidelity monitoring tools and the incorporation of objective measures to enhance both data reliability and intervention adherence.

## Conclusions

In conclusion, this cluster-randomized controlled trial provided the evidence that a comprehensive, school-delivered intervention incorporating health education, physical activity promotion, and family engagement components was associated with reductions in BMI metrics and obesity rates in Chinese children aged 8–10 years. While these findings demonstrated the program’s efficacy under controlled conditions, we should explore its scalability in diverse age groups and different regions to assess broader applicability in further study.

## Supplementary Information

Below is the link to the electronic supplementary material.

Supplementary Material 1

## Data Availability

The data supporting the conclusions of this article are included within the article. Please contact the corresponding author if someone wants to request the data from this study.
