# Supplementary material for: Multi-component school intervention reduces obesity and improves health behaviors in children: a cluster- randomized controlled trial
Source: Sci Rep. 2025 Nov 18;15:40607. doi: 10.1038/s41598-025-24295-y (PMC12627419; doi:10.1038/s41598-025-24295-y)
Supplement: Supplementary file 1 — Supplementary Material 1 [file 41598_2025_24295_MOESM1_ESM.docx]

**Table 1. Description of the Multifaceted Intervention Components**

| **Intervention components** | | **Who delivered** | **When delivered** | **What delivered** |
| --- | --- | --- | --- | --- |
| **Three components targeting children** | | | | |
| 1. Health education | | Trained class teachers | Every 2 to 3 weeks (10 sessions§ ) | Five core messages: two “*NOT*”, two “*LESS*”, and one “*MORE*” messages* were lectured on class |
| 2.physical exercise reinforcement | | Trained physical education teachers | Every school day | One-hour moderate-to-vigorous-intensity physical activity per day within school |
| 3.BMI monitoring and feedback | | Trained health care teachers | Monthly | Monitoring body weight and height of the children, and providing feedback on BMI status and changes |
|  |  | Children | Weekly | Measuring body weight only |
| **Two components targeted the children's environment** | | | | |
| schools | 1.School policies supporting obesity prevention | Trained school teachers^&^ | Every school day | ♆Not selling, eating, or buying unhealthy snacks or sugar-sweetened beverages within school |
|  | 2. Health education for school teachers | Trained project staff | In the first month (1 session) | Five core messages |
| families | 1. Health education for parents | Trained project staff | In the start and halfway of the 1st semester, in the start of the 2nd semester (3 sessions) | Five core messages; feedback on children’s BMI and behaviors through app |
|  | 2. Reinforcement of children’s physical activity outside school | Parents | Every day | Supervising and encouraging children to perform physical activities outside of school |
|  | 3. Supporting children to manage body weight | Parents | Weekly | Recording and tracking diet and physical activity behaviors of the children (weekly) through app |
|  |  |  | Monthly | Tracking BMI of the children (monthly) through app |

*NOT eating excessively; NOT drinking sugar-sweetened beverages; LESS high-energy food; LESS sedentary time; MORE physical activities. & School teachers included school principals, class teachers, health care teachers, and physical education teachers. ♆ “Not selling”: Not selling unhealthy snacks or sugar-sweetened beverages within school; “Not eating”: Telling students not to eat unhealthy snacks or drink sugar-sweetened beverages within school; “Not buying”: Students being educated by class teachers not to buy unhealthy snacks or sugar-sweetened beverages around school.

**Table 2. Measurements and Their Associated Outcome Variables**

| **Measurements** | **Time points** | | | **Instrument** | **Number of measures at each time point** | **Method of assessment** | **Outcome variables** |
| --- | --- | --- | --- | --- | --- | --- | --- |
|  | **Baseline** | **4**  **months** | **9**  **months** |  |  |  |  |
| **Adiposity** | | | | | | | |
| Height | Yes | Yes | Yes | Stadiometer (Huateng GMCS-1) | Twice (third measure if difference > 0.5 cm)^1^ | Measured to the nearest 0.1 cm | BMI(together with children’s sex and date of birth), prevalence of overweight or obesity, prevalence of obesity, (BMI status was defined according to Chinese national screening criteria |
| Weight | Yes | Yes | Yes | Lever scale (Wujin RGT-140) | Twice (third measure if difference > 0.1 kg)^2^ | Measured to the nearest 0.1 kg |  |
| Body fat percentage | Yes | No | Yes | Body component instrument (Tanita MC-780 MA) | Once | According to instructions of the instrument | Body fat percentage |
| Waist circumference | Yes | Yes | Yes | Tape (MyoTape) | Twice (third measure if difference > 1.0 cm)^3^ | Measured to the nearest 0.1 cm | Waist circumference, waist-to-hip ratio |
| Hip circumference | Yes | Yes | Yes | Tape (MyoTape) | Twice (third measure if difference > 1.0 cm)^3^ | Measured to the nearest 0.1 cm |  |
| **Physical activity and dietary behaviors** | | | | | | | |
| Stage of behavior  change for weight  management | Yes | No | Yes | The validated items measuring stages (in the action stage versus in the pre-action stage) of  behavior change for the purpose of weight management | Once |  | Percentage of children in the action stage of behavior change for weight management (children actually being initiated come behavioral change, in comparison with those in the pre-contemplation (i.e., not thinking about becoming engaged in the behavior change) or contemplation (i.e., not involved in the behavior change but was considering getting involved in the behavior in the near future) stage) |
| Dietary behavior | Yes | No | Yes | An updated version of a  previously validated Block Kids Food Screener questionnaire  ; previously validated  Children Eating Behavior Questionnaire | Once | Children finished the  questionnaires in the classroom in the presence of the trained outcome assessors who can provide guidance and help. | Percentage of children who did not drink sugar-sweetened beverages, percentage of children who did not eat high-energy food (fried food, western fast food), excessive eating behavior (scores of satiety responsiveness, scores of emotional over-eating scores) |
| Screen viewing  behavior | Yes | No | Yes | An updated version of a  previously validated screen viewing questionnaire | Once |  | Time spent on screen viewing |
| Self-reported moderate-to  vigorous physical  activity | Yes | No | Yes | An updated version of a  previously validated Youth Risk Behavior Survey questionnaire | Once | Children finished the  questionnaires in the classroom in the presence of the trained outcome assessors who can provide guidance and help | Number of days performing moderate-to-vigorous physical activity ≥1 hour per week (this cut-off was defined based on “Global Recommendations on Physical Activity for Health” ) |
| **Obesity-related knowledge** | | | | | | | |
| Obesity-related  knowledge | Yes | No | Yes | Items designed based on the key messages of health education activities^4^ | Once | Children finished the  questionnaires in the classroom in the presence of the trained outcome assessors who can provide guidance and help. | Scores of obesity-related knowledge |
| **Physical fitness** | | | | | | | |
| One-minute rope jump | Yes | No | Yes | Not applicable | Once | Measured to unit of number | Number of rope jumps within one minute |
| One-minute sit-up | Yes | No | Yes | Not applicable | Once | Measured to unit of number | Number of sit-ups within one minute |
| Long standing jump | Yes | No | Yes | Not applicable | Third^5^ | Measured to the nearest 1 cm | Distance of long standing jump |
| Shuttle run (50 m×8) | Yes | No | Yes | Not applicable | Once | Measured to the nearest 0.1 s | Duration of shuttle run (50 m×8) |

1 Where two values were ≤ 0.5 cm, a definitive measurement value was calculated as the average of the two. For individuals with three values recorded, a definitive measurement value was calculated as average of the closest pair or average of all three readings (if there were no two closest readings).

2 Where two values were ≤ 0.1 kg, a definitive measurement value was calculated as the average of the two. For individuals with three values recorded, a definitive measurement value was calculated as average of the closest pair or average of all three readings (if there were no two closest readings).

3 Where two values were ≤ 1.0 cm, a definitive measurement value was calculated as the average of the two. For individuals with three values recorded, a definitive measurement value was calculated as average of the closest pair or average of all three readings (if there were no two closest readings).

4 For example, children were asked "*Is it correct that drinking sugar-sweetened beverage cannot substitute drinking water*?" and three choices were provided (*“correct”; “wrong”; “unknown”*). Children who chose “correct” would be given 1 score, and those choosing “wrong” or “unknown” would be given 0 score.

5 A definitive measurement value was obtained from the maximum of the three measurements.

**Table 3. Comparison of Children Included in the Primary Outcome Analysis (n=396) and Those Lost to Follow-up (n=4) by Baseline Characteristics**

|  | **Children included in primary outcome analyses**  **(n=396)** | **Children without follow-up assessment**  **(n=4)** |
| --- | --- | --- |
| **Group allocation, n (%)** |  |  |
| Intervention group | 198(50.0) | 2(50.0) |
| Control group | 198(50.0) | 2(50.0) |
| **Sex, n (%)** |  |  |
| Boys | 198(50.0) | 1(25.0) |
| Girls | 198(50.0) | 3(75.0) |
| **Primary caregiver (parents), No (%)** | 275(69.4) | 4(100.0) |
| **Maternal educational level (above high school), No (%)** | 205(51.8) | 3(75.0) |
| **Weight status (overweight/obesity), No (%)** | 163(41.2) | 1(25.0) |
| **Age, mean (SD), y** | 9.14(0.38) | 9.50(0.58) |
| **Height, mean (SD), cm** | 138.26(6.36) | 133.6(1.86) |
| **Weight, mean (SD), kg** | 35.41(8.76) | 30.25(5.53) |
| **BMI, mean (SD), kg/m^2^** | 18.3(3.59) | 16.86(2.65) |
| **Waist circumference, mean (SD), cm** | 63.96(10.83) | 59.7(7.33) |
| **Hip circumference, mean (SD), cm** | 74.85(9.60) | 71.58(6.14) |
| **Body fat percentage, mean (SD)** | 19.64(10.71) | 16.37(7.00) |
